# Prediction of top round beef meat tenderness as a function of marinating time based on commonly evaluated parameters and regression equations

**DOI:** 10.1002/fsn3.2454

**Published:** 2021-07-07

**Authors:** Mohammadreza Rostamani, Homa Baghaei, Marzieh Bolandi

**Affiliations:** ^1^ Department of Food Science and Technology Damghan Branch Islamic Azad University Damghan Iran

**Keywords:** marinating time, natural tenderizer, regression equations, tenderness

## Abstract

This study investigates the influence of 24‐hr marination (with different plant extracts and vinegar) at refrigerated conditions on commonly evaluated physicochemical and textural attributes, including pH, water‐holding capacity (WHC), collagen solubility, moisture, drip loss, and shear force values of beef meat. The results reflected the appropriate correlation between each pair and indicated the efficiency of the household marination procedure to acquire more palatability and tender beef meat. Therefore, to predict beef meat tenderness by applying the Warner–Bratzler shear force (WBSF), a strong positive correlation with the drip loss (*p* < .01) and a notable negative correlation with the moisture content (*p* < .01) emphasized the importance of moisture improvement and shear force reduction in affecting tenderness of baked beef meat. The regression equations and R‐squared values were revealed the favorable correlation between collagen solubility and WHC (*y* = 0.1035*x*−0.8431, *R* ^2^ = .98) as well as moisture and WBSF (*y* = −0.3297*x* + 102.58, *R* ^2^ = .99) in marinated beef meat. Electrophoresis patterns of isolated myofibrillar proteins disclosed remarkable degradation of myosin heavy chain (MHC), desmin, actin, and tropomyosin during the first day of aging. The noticeable ultrastructural destruction and connective tissue solubilization were observed by microscopy images. These outcomes were a good tenderness predictor be utilized in retailing and industrial scale.

## Graphical abstract

The results reflected the appropriate correlation between each pair and indicated the efficiency of the household marination procedure to acquire more palatability and tender beef meat. These outcomes of this study were a good tenderness predictor be utilized in retailing and industrial scale.

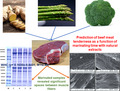

## 1 INTRODUCTION

Tenderness is greatly impacted the organoleptic and quality characteristics of meat and its products (Bojorges et al., 2020; Brito Lopes et al., 2020; Ma & Kim, 2020). Inconsistency and nonuniformly texturized meat products are the major problems encountered by the meat industry that affecting tenderness and also customer satisfaction (Stadnik & Dolatowski, 2011; Uyarcan & Kayaardi, 2019; Zhou et al., 2020). Hence, over the past few decades, the industrial sections concentrated on discovering safe and innovative ingredients and methods to solve this issue. In aged animals, firmness is accompanied by the development of perimysium, endomysium thickness, and the generation of cross‐links between collagen molecules. Overtenderization or lack of softness in different parts of meat chunks mainly emerged in raw meat with high connective tissues within postmortem conditioning by traditional components, mechanical and electrical procedures (Bhat et al., 2018; Lang et al., 2020).

The conventional and technological interventions are currently established by endogenous meat proteases and plant‐derived exogenous proteases (Bojorges et al., 2020; Chang et al., 2021). As the demand for consuming meat and its products increase in densely populated and developing countries, it is necessary to employ promising compounds to create quick and uniform tenderness in meat chunks without many destructive effects on myofibrillar and connective tissues (Arsalane et al., 2019; Noshad et al., 2021). Since the addition of these components is dependent on their hydrolytic activities toward targets, the dose exceeding the limit may affect the quality and texture (Dhital & Vangnai, 2019; Wang et al., 2020). Despite the limitations and undesired results caused by utilizing natural materials in different meat and cuts such as mushy texture and bitterness, the usage of them is well controlled through monitoring the alteration trend. Further, these inartificial substances are eco‐friendly and cost‐effective. Multiple kinds of research have been considered to elucidate the mechanism of the proteolytic activity of broccoli (Sun et al., 2016; Wang et al., 2004), green asparagus (Ha et al., 2013; Mazaheri Kalahrodi et al., 2020), ginger (Ha et al., 2012; He et al., 2015; Sullivan & Calkins, 2010; Tsai et al., 2012), soy sauce (Kim et al., 2013), and vinegar (Mazaheri Kalahrodi et al., 2020; Żochowska‐Kujawska et al., 2017), individually or simultaneously.

The literature review indicated that the prominent protease of both juice and extract of broccoli (*Brassica oleracea var. Italica*), green asparagus (*Asparagus Officinalis* L.), and ginger (*Zingiber Officinale*) is cysteine protease belonged to the papain family with the proteolytic activity of 16.9, 14.1, and 16.6 U/g, respectively (Sun et al., 2016). The optimal temperatures for the activity of their proteases are at below 18–20, 40–45, and 60°C assigned to broccoli, asparagus, and ginger, whereas acidic, neutral–slightly alkaline, and acidic ranges were optimum pH values, respectively. The molecular weight of their proteases is approximately 45–70 kilodalton (kDa) (broccoli), 28 kDa (asparagus), and 23–32 kDa (ginger). Consequently, their proteolytic activity on contractile, structural, regulatory as well as connective proteins, that is, actomyosin, desmin, tropomyosin, and collagen, were contributed to generate more tender meat (Bhat et al., 2018; Zhou et al., 2020).

The application of weak organic acids and salts not only can be led to pleasant flavor, but also can be useful in promoting the technological worth of meat during production. The occurrence of these changes might be related to the increase of trapped free water in capillary tubes, which were developed under the influence of acidic and enzymatic treatments (Dhital & Vangnai, 2019; Mazaheri Kalahrodi et al., 2020).

Nonetheless, no information on using different ingredients as a marinade mixture was acquired to complete findings in this regard. Accordingly, we are supposed to apply the combination of natural extracts and acidic conditions to modify meat structure during storage; so, their effects on the quality of marinated meat can be evaluated by ordinarily physicochemical and structural parameters. Also, their relevance might be predicted by regression equations. Therefore, this research aimed to predict the tenderness of marinated top round beef meat as a function of marinating time based on commonly evaluated parameters and regression equations.

## 2 MATERIALS AND METHODS

### 2.1 Materials

The top round beef (*semitendinosus* muscles, breed: Holstein; sex: male; age: nearly 8–10 months old) was obtained from a slaughterhouse in Gorgan, Iran. Fresh broccoli (*Brassica oleracea* L.), green asparagus (*Asparagus Officinalis* L.), ginger (*Zingiber Officinale*), soy sauce, and white vinegar (Kikkoman brand, Walworth) were purchased from the local market (Gorgan, Iran). All the chemicals and reagents were of analytical grade (Merck Chemicals Co., Darmstadt, Germany; Sigma‐Aldrich Co.).

### 2.2 Preparation of broccoli, asparagus, and ginger extracts

The crude extracts were prepared following the technique described by Amid et al. (2012) with slight modifications. First, fresh plants were ground by a mixer (D70, Moulinex, Germany), and then 10 g of each sample was blended with 100 ml of sodium phosphate buffer (0.1 M, pH = 7.5) at 4°C for 2 min. This mixture was filtered through cheesecloth and centrifuged (Beckman Coulter centrifuge, 5XP, Mervue) at 15,000 × *g* at 4°C for 15 min. The resulting supernatants were recovered and kept at −40°C until further use.

### 2.3 The marination procedure

All visible fat and connective tissues were trimmed from beef muscles. The fat‐free beef muscles were cut into uniform slices of 2 × 2 × 2 cm^3^. Thirty pieces of meat were used for each treatment. The marinade mixture formula was optimized using response surface methodology (RSM) to obtain the proper contribution of each ingredient in the marinade mixture. Then, the specimens were sprayed with the marinade mixture (30% v/w, pH = 4.89), including the formula presented in Table 1. Subsequently, all treated samples were sealed in low‐density polyethylene bags and stored at 4°C for 24 hr. The nonmarinated sample was determined as the control group in advance. To evaluate the role of marinating time on dependent parameters and to predict their relationships, the responses were assessed in every specified time of 0‐, 3‐, and 24‐hr intervals.

**TABLE 1:** The optimum formula of the marinade mixture was applied in the current research

Ingredients	Quantity (%v/w)
Broccoli extract	26.5
Asparagus extract	13.52
Ginger extract	26.26
Soy sauce	3.68
White vinegar	5
Distilled water	25.04

### 2.4 pH determination

Five grams of raw sample was homogenized with 50‐mL chilled distilled water. After homogenization, the sample was centrifuged at 8,000 × *g* for 60 s. The pH was determined using a digital pH meter (Crison 507) with a calibrated electrode specific for meat/meat products (Kim et al., 2013).

### 2.5 WHC

The WHC was made according to the technique proposed by Naveena et al. (2004). Minced meat (20 g) was centrifuged with 30 ml of 0.6 M NaCl. The mixture was blended for 1 min and kept at 4°C for 15 min. Then, it was re‐mixed and again centrifuged at 3,000 × *g* (R‐24, Remi Instruments, India) for 25 min. The resulting supernatant was measured, and the WHC was represented in percentage.

### 2.6 Collagen solubility

The method described by Mahendrakar et al. (1989) was carried out to measure collagen solubility. Five grams of the treated sample was taken in a 250‐mL beaker and immersed in a water bath of 100°C for 30 min. After taking the baked sample out of the beaker, they cut into small cubes and homogenized with 50‐mL distilled water at chilled temperature for 2 min. Next, the extract was centrifuged at 1,500 × *g* for 30 min. A small fraction of baked out extract was hydrolyzed at 108°C for 18 hr to determine the percentage of solubilized hydroxyproline. The solubility of collagen was calculated as follows:

Collagen solubility (%)=7.14×solubilized hydroxyproline (%)

### 2.7 Moisture content

The moisture content of all samples was measured via 12 hr of losing weight at 105°C in a hot‐air oven (SW‐90D, Sang Woo Scientific Co.) following the instruction as outlined by AOAC (Mazaheri Kalahrodi et al., 2020).

### 2.8 Mechanical texture measurement

The Warner–Bratzler shear force values of the treated samples were measured using an Instron (4301, Universal device) after chilling baked samples at an ambient temperature. The Warner–Bratzler cutting apparatus was equipped with 1‐mm‐thick flat blade attachment samples and were cut at right angles parallel to the muscle fibers orientation. The speed was set to 50 mm/min. A load cell of 1 KN was utilized. The required force for shearing the specimens was reported in Newton (Burke & Monahan, 2003).

### 2.9 Drip loss percentage

The drip loss percentage was computed from differences between the initial and ultimate weight of suspended samples during 24 hr of storage at refrigerated condition (Mazaheri Kalahrodi et al., 2020).

### 2.10 One‐dimensional sodium dodecyl sulfate–polyacrylamide gel electrophoresis (SDS–PAGE)

Separation of the myofibrillar protein fragments was carried out based on the technique previously presented by Sikes et al. (2010). The Biuret method was used to determine the protein concentration of the separated myofibrillar faction (Gornall et al., 1949). To perform the SDS–PAGE process described by Laemmli (1970), Bio‐Rad's vertical electrophoresis (PowerPac™ Basic model) was used with the aid of 12% running gels and 5% stacking gels (20 μl loaded). Coomassie Brilliant Blue R250 (B7920, Sigma) was applied to stain the loaded gel that was subsequently destained by a solution at a ratio of methanol (50): distilled water (40): acetic acid (10). The protein bands were identified in comparison with a standard protein marker (Precision Plus Protein Standards, Bio‐Rad Lab.).

### 2.11 Scanning electron microscopy (SEM) images

The ultrastructural properties of all samples were scrutinized by the strategy attributed to Naveena et al. (2011). The meat thickness of approximately 2–3 mm was prepared. Then, they were fixed in two stages. First, they were fixed with 2.5% glutaraldehyde and 0.1 M phosphate buffer (pH = 7.2) at 4°C for a day. Second, they were fixed by 2% osmium tetroxide and H_2_O (Sigma, USA) and dehydrated by different ethanol dilutions. Afterwards, they were cut using a razor blade in liquid nitrogen and were coated by a thin layer of gold. Finally, they were scanned by SEM (S‐3000N) at a magnification level of ×1,000.

### 2.12 Statistical analysis

All experiments were conducted twice, and all the analyses were performed in triplicate. The statistical analysis was performed using the Statistical Package for the Social Sciences (SPSS Inc., version 23.0, IBM). The results were subjected to analysis of variance (ANOVA). The significance level was set at *p* < .05. The Pearson's correlation coefficient (*R*) was used to determine the correlation between each pair of defined parameters, that is, pH versus WHC, pH against collagen solubility, collagen solubility versus WHC, moisture versus WBSF, drip loss versus moisture, and drip versus WBSF values. Also, the linear regression model was conducted, and the coefficient of determinations (*R* ^2^) was calculated between the aforementioned attributes. Also, correlation is significant at *p* < .05 and *p* < .01.

## 3 RESULTS AND DISCUSSION

### 3.1 Effect of marinating time on functional and textural attributes of beef meat exposed to the marinade mixture

Enzymatic modification of proteins remarkably improves their technological characteristics such as WHC, emulsifying stability, and production yield through increasing the protein dissolvability. The degree of meat tenderness is chiefly dependent on the degree of weakness and breakdown of myofibrillar organization and soluble protein content, which is straightly linked to produce uniformly texturized meat products and modifying the folding and shearing quality of them (Aminlari et al., 2009). Briefly, based on the observations that came from the current research, the technological and textural properties (will be mentioned below) pointed out the suitable efficacy of marinade treatment applied over time. Upon aging at refrigerated condition on the first day, the mean pH values were gradually decreased (*p* < .05) from the beginning of the marinating procedure until its completion. The mean value and standard deviation of pH measured for unmarinated sample at the beginning of storage time were 6.15 ± 0.42. In contrast, the values of 5.74 ± 0.25, 5.68 ± 0.36, and 5.65 ± 0.37 were reported for marinated samples in the 0th, 3th, and 24th hr of storage, respectively. No evidence of a statistically significant difference was acquired between 3 hr and 24 hr of marinating time in pH determination (*p* > .05), while a statistically significant difference in pH mean values was observed between the early moments after marinating and the other times (0 hr and 3–24 hr, *p* < .05). The mean value and standard deviation of WHC reported for the control sample at the beginning of storage time were 26.2 ± 1.7. As the conditioning time progressed, the increase of WHC accelerated (*p* < .05). At the end of the storage period, the ultimate WHC mean values of 30.8%, 32.4%, and 35.6% were assigned to 0, 3, and 24 hr of storage, respectively. Similarly, as prolonged exposure of marinated samples to the marinade mixture, the collagen solubility mean values increased compared to nonmarinated specimens (*p* < .05). This trend continued until the end of storage, particularly in the 24th compared to the 3rd hr of aging. Nonetheless, no significant changes in collagen solubility were observed between 3 hr and 24 hr of marinating time in treated samples (*p* > .05). The initial moisture value reported for the control group was 76.8%. At the same time, the mean value of moisture remained unchanged for the marinated sample (0 hr, *p* > .05), while a considerable incremental trend (*p* < .05) was occurred from the 3rd to 24th hr of the aging period (3–24 hr, *p* < .05). The ultimate moisture mean value was reached 82.6% on the final day of refrigerated storage. The reverse trend was recognized for drip loss and WBSF values subsequently confirmed by myofibrillar protein patterns and microscopic detections, with increasing marinating time up to 24 hr, a significant (*p* < .05) reduction in drip loss values along with a corresponding decrease in the WBSF was recorded. The original mean values of 4.11% and 79.15 Newton were expressed for drip loss and WBSF of the control sample, respectively. In the marinated sample, these values were reached 3.65% and 60.81 Newton, respectively (*p* < .05, 24 hr). The Pearson's coefficient of correlations among physicochemical and textural characteristics, that is, pH, WHC, collagen solubility, moisture, drip loss, and WBSF mean values of marinated beef meat, are presented in Table 2 to show the relevance between each pair of mentioned parameters. The R values of −0.92, −0.95, and 0.99 were determined between each couple of pH value, WHC, and collagen solubility percentages (Table 2). As shown in Table 2, pH is negatively correlated with WHC and collagen solubility with remarkable discrepancy for the latter (*p* < .05). In contrast, the differences between the former, that is, pH and WHC value, are insignificant at both levels of probability (*p* > .05 and *p* > .01). WHC has positively correlated with collagen solubility, and indicated a strong, direct, and noticeable relationship between these two sets of attributes (*p* < .01). The relationship between moisture content against drip loss percentage as well as moisture values versus WBSF in Newton was reverse with the very high negative correlation coefficient of −0.98 and −0.99 at the significant level of *p* < .05 and *p* < .01, respectively, whereas a positive linear correlation (*R* = .99) was recognized between two parameters of drip loss and WBSF values (*p* < .01, Table 2). The other comparisons were not significant at both levels of probability (*p* > .05 and *p* > .01, Table 2).

**TABLE 2:** The Pearson's coefficient of correlation (*R*) among physicochemical and textural characteristics of marinated beef meat

Parameter	pH	WHC	Collagen solubility	Moisture	Drip loss	WBSF
pH	–	−0.92^NS^	−0.95*	−0.64^NS^	0.76^NS^	0.70^NS^
WHC	–	–	0.99**	0.85^NS^	−0.91^NS^	−0.89^NS^
Collagen solubility	–	–	–	0.83^NS^	−0.91^NS^	−0.88^NS^
Moisture	−0.64^NS^	0.85^NS^	0.83^NS^	–	−0.98*	−0.99**
Drip loss	0.76^NS^	−0.91^NS^	−0.91^NS^	–	–	0.99**
WBSF	0.70^NS^	−0.89^NS^	−0.88^NS^	–	–	–

*Correlation is significant at the 0.05 level (*p* < .05).

**Correlation is significant at the 0.01 level (*p* < .01).

Abbreviation: ^NS^, not significant.

**FIGURE 1:**
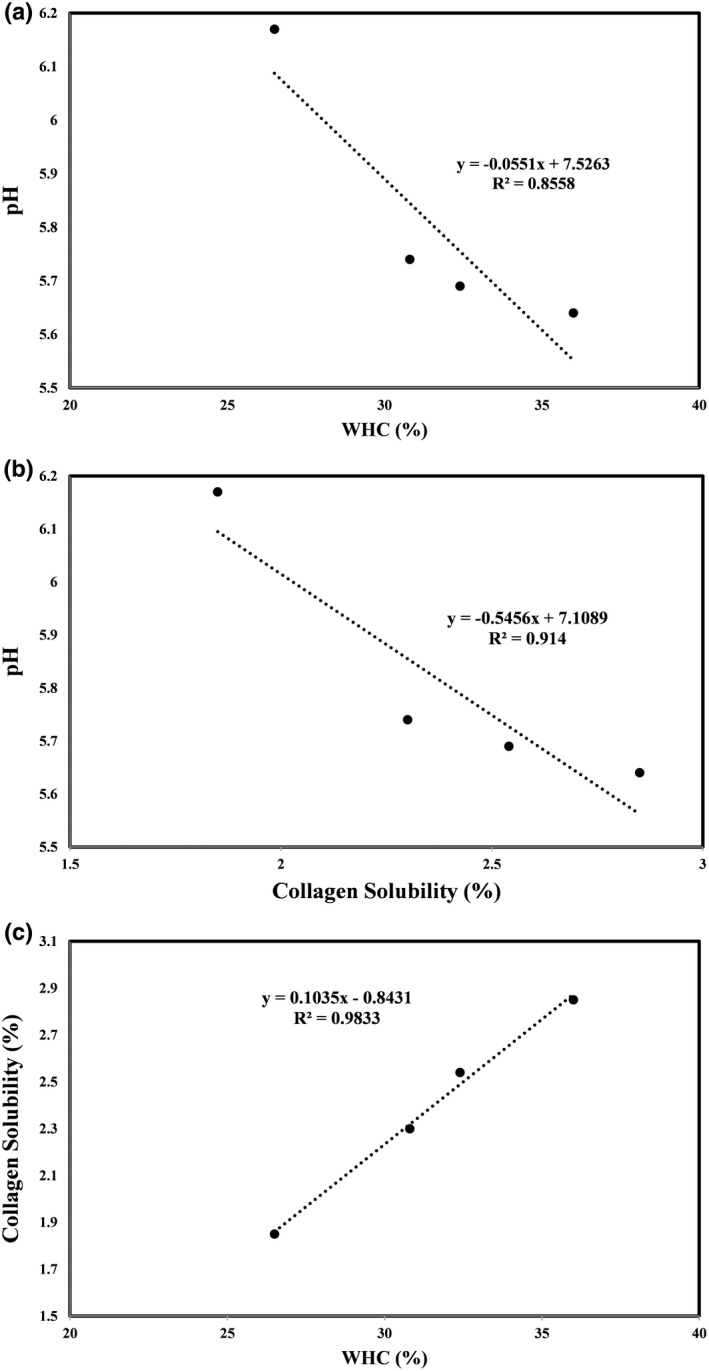
Correlation of pH versus WHC (a), pH versus collagen solubility (b), and collagen solubility versus WHC (c) of all treated beef meat

**FIGURE 2:**
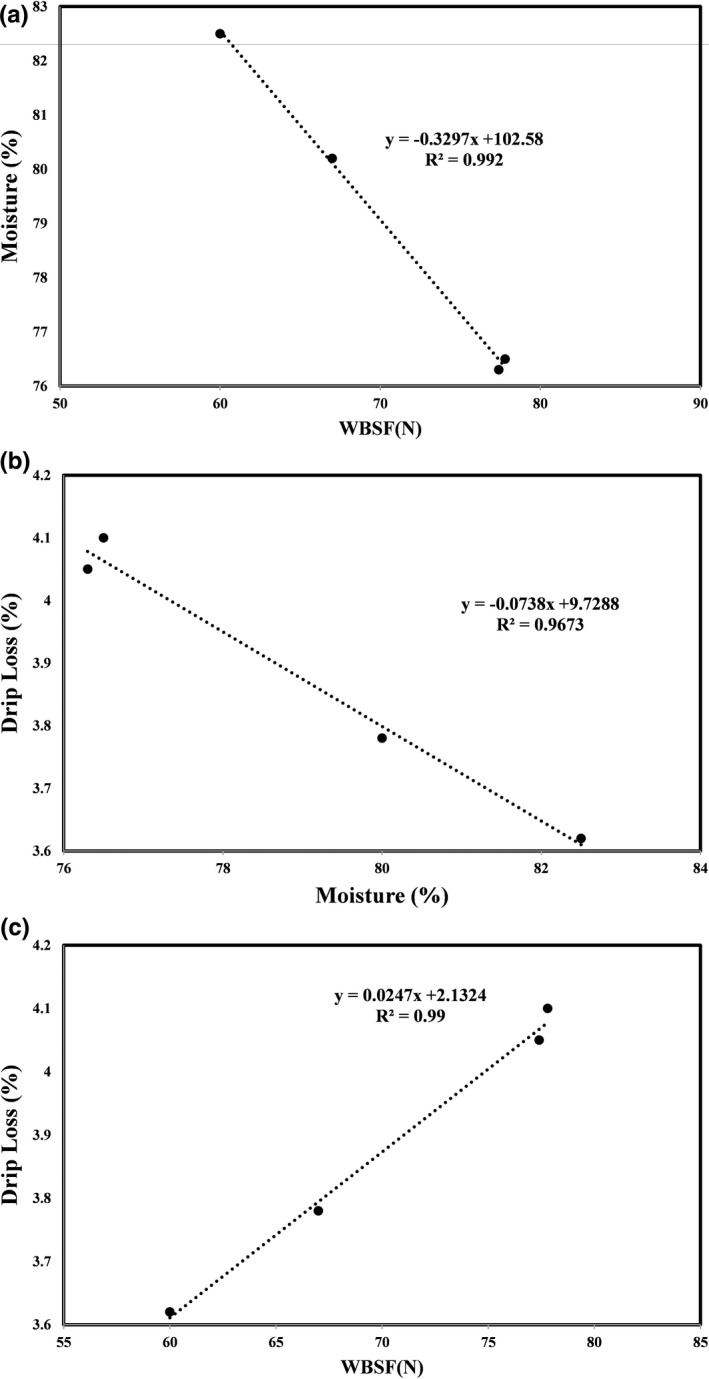
Correlation of moisture versus WBSF (a), drip loss versus moisture (b), and drip loss versus WBSF (c) of all treated beef meat

The incremental evolution of dissolve proteins in muscles influences the structural properties. Thus, this structural alteration can be fulfilled by chemical and enzymatic modifications (Maqsood et al., 2018). As illustrated in Figures 1 and 2, graphs are plotted to exhibit the correlation between each pair of considered parameters, including pH, WHC, collagen solubility, moisture, drip loss, and WBSF values in all samples. In this context, mathematical equations, which are prepared by the regression method, are a quick and easy device for evaluating and predicting the correlation between variables, so when a parameter is provided, the other one is specified via these equations. The correlation of pH versus WHC, pH against collagen solubility, and collagen solubility versus WHC values are depicted in Figure 1a–c, respectively. The regression equations and *R*‐squared of *y* = −0.0551*x* + 7.5263 (*R* ^2^ = 0.85), *y* = −0.5456 + 7.1089 (*R* ^2^ = .91), and *y* = 0.1035*x*−0.8431 (*R* ^2^ = 0.98) were referred to correlation between pH and WHC, pH and collagen solubility as well as collagen solubility and WHC, respectively (*p* < .05). Obviously, the strongest linear relationship indicated a good regression was observed between two sets of collagen solubility and WHC, which is well appeared in SEM images (will be explained later). As WHC increased, the soluble collagen enhanced as well. These results were the same considered in different kind of independent variables, that is, red/white meat, marinade type, marinating time, and temperature by numerous researches conducted by Pawar et al. (2007), Yusop et al. (2010), Naveena et al. (2011), Gokoglu et al. (2017), Maqsood et al. (2018), Kadıoğlu et al. (2019), and Mazaheri Kalahrodi et al. (2020). Besides, lower pH caused higher WHC and soluble collagen in treated specimens over time. This could be due to the impact of marinade ingredients as well as their concentration on the ionic power and environmental acidity degree. Moreover, the preservation role of marinating with marinade mixture in extended cold storage against the growth of microbes may decrease the pH level (Cheok et al., 2011). Glycogen resources and their participation in the glycolysis process may be another reason for lowering pH in marinated specimens (Mazaheri Kalahrodi et al., 2020). WHC is the ability of meat to hold inherit or absorbed water among the filaments' spaces. Therefore, it is controlled by multi‐parameters such as physiological (different breed and genotype), anatomical (the type of muscle), slaughtering, and processing conditions (aging condition, methods of analysis, and process of homogenization) (Aminlari et al., 2009). The pH of meat is one of the most influential factors on its tenderness (Chaurasiya et al., 2015; Santos et al., 2020). Since, pH straightly affects the number of negative charges on the protein molecules that can bond to water molecules, pH values below or above the isoelectric point can expand space in the muscle filaments through hydrophilic properties enhancement. Thus, improvement in the WHC percentage would occur. Following that, marinated samples keep a high amount of water, and the baked samples demonstrate increased levels of juiciness and tenderness (Pawar et al., 2007). Likewise, under the acidic conditions provided by soy sauce and vinegar, the collagen molecule is susceptible to hydrolysis by different types of proteases which consist of natural meat proteases (calpains and cathepsins) and added proteases (cysteine and aspartic proteases of broccoli, asparagus, and ginger extracts). Thereby, collagen is converted to gelatin by applying a baking temperature (Ayudiarti et al., 2020). Hence, the enhanced tenderness could be understood from the solubilized collagen and the decrease insoluble tissues in marinated samples (Maqsood et al., 2018). The sentence “Hence, the enhanced tenderness…in marinated samples” lacks clarity. Please check. Lastly, in this study, the sufficiency of exposure to marinade could be explained by increases in WHC and collagen solubility due to protonation of carboxyl groups in muscle proteins and swelling of connective proteins. These results were in agreement with findings of Kim et al. (2013).

Increased marinade intake and reduction of removed moisture in meat samples have economic advantages and higher final production yield in meat products. These products are generally found to be juicier and softer. As displayed in Figure 2a–c, each pair of three variables, that is, moisture and WBSF (a), drip loss and moisture (b), and drip loss and WBSF (c), were regressed to understand whether determining equations are a good fit to predict the connection between these commonly evaluated factors. The regression equations and R‐squared of *y* = −0.3297*x* + 102.58 (*R* ^2^ = .99), *y* = −0.0738*x* + 9.7288 (*R* ^2^ = .96), and *y* = 0.0247*x* + 2.1324 (*R* ^2^ = .99) were associated with the strong correlation between moisture and WBSF, drip loss and moisture, and drip loss and WBSF, respectively. Regarding their R‐squared values, these outcomes were a good tenderness/toughness predictor in all treated samples. The reverse linear correlation between drip loss, moisture, and WBSF indicated that lower WBSF and drip loss could be linked to the higher moisture content in marinated specimens. This phenomenon might be due to the penetration of marinade mixture to the interior space of meat structure, following that, covalent attachment between the key myofibrillar proteins, that is, actin, myosin, and water molecules, may cause more water to maintain inside meat texture through the water immobilization. Thereby, more tenderness and juiciness will occur in marinated beef meat by reducing shearing force and drip loss (Cheok et al., 2011). The consequence of this structural alteration is moisture enhancement, which is well verified by their very high R‐squared values. This observation could be corroborated by the evidence on proteolysis action of proteases caused by broccoli, asparagus, and ginger extracts, which could modify marinated meat structure compared to the control sample. These findings were in line with the outcomes of research conducted by Tsai et al. (2012). They found that the significant destruction of desmin proteins prevents the effective transmission of myofibrils contraction into the entire cell, and thus, more moisture will remain inside the tissue. In this study, drip loss was affected by the conditioning time (*p* < .05). The gradual breakdown of desmin proteins from the beginning of cold storage up to the end supported by electrophoresis results (will be discussed thereafter) caused a noticeable reduction of drip loss in all the samples. In conjunction with data obtained in this research, reduction of drip loss in beef and pork meat chunks treated with several ingredients has been expressed by Sultana et al. (2008) and Yang et al. (2006), respectively. Meanwhile, a notable reduction of WBSF and energy to the peak rates in beefsteak marinated with the combination of asparagus juice and balsamic vinegar during 48 hr of storage was declared by Mazaheri Kalahrodi et al. (2020), due to the cysteine protease activity of asparagus juice, which is activated in low pH conditions provided by balsamic vinegar. Moreover, they pointed to the destruction of MHC, desmin protein, and connective tissue as a function of marinade type and time to demonstrate a more tender beef steak. Similarly, Kadıoğlu et al. (2019) admitted the interaction effect of time and enzymatic treatment to reduce the shear force and further decomposition of myofibrillar proteins to enhance meat tenderness. Based on the evidence of evaluated parameters in the current research, the most technological and textural alterations as affected by the independent variable, that is, marinating time, were observed within the first 24 hr of storage, which was in accordance with the data informed by Rajagopal and Oommen (2015) in buffalo meat. Besides, the diminishing trend for WBSF values over 8 days of storage has been considered by them. This structural modification and decrease in the shearing force of marinated samples could be attributed to the type, concentration, and proteolytic activity of marinade ingredients as well as natural meat proteases including calpains, lysosomal cathepsins, and multicatalytic proteinase complex, which play a profound role in the weakening of muscle protein and solubility of collagen.

### 3.2 Effect of marinating time on electrophoretic patterns of myofibrillar proteins of beef meat exposed to the marinade mixture

**FIGURE 3:**
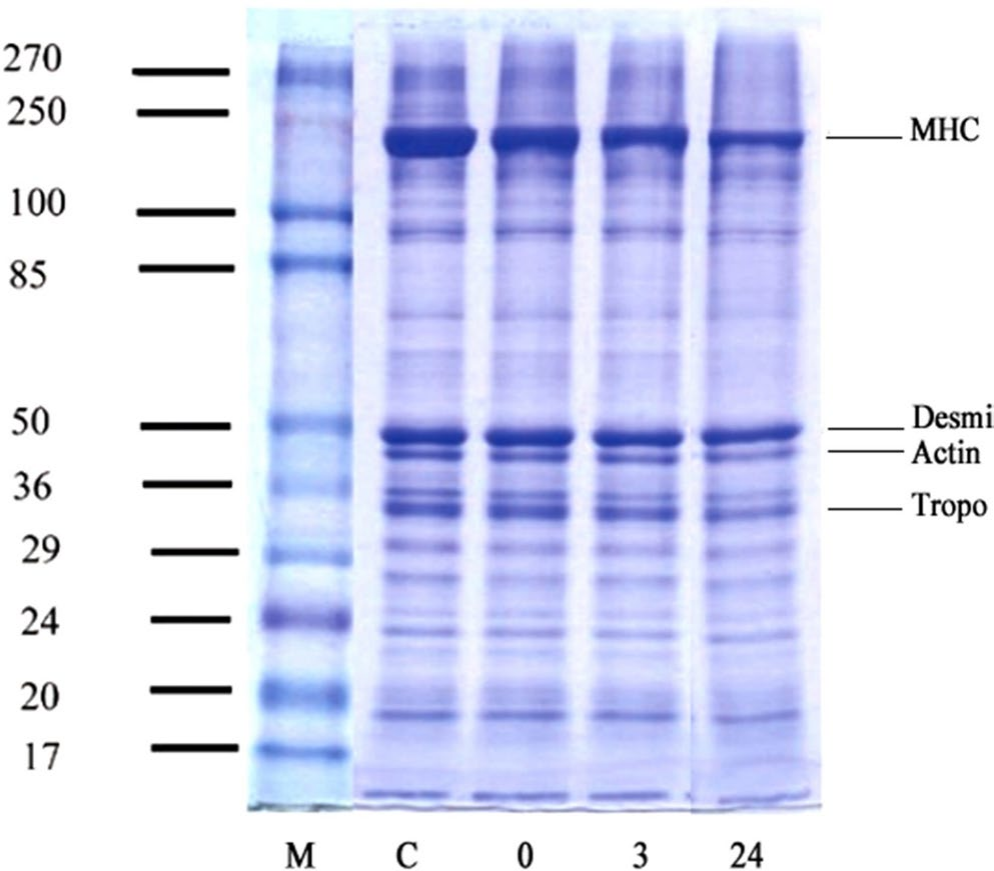
SDS‐PAGE image of isolated myofibrillar proteins of beef meat cuts marinated within 24 hr of marinating time. Treatments: MHC; Myosin heavy chain, Tropo: Tropomyosin, M: Standard protein marker, C: unmarinated sample, the other columns indicated marinated samples within 0, 3, and 24 hr of storage at cold condition, respectively

The effect of marinating time on electrophoretic patterns of myofibrillar proteins segments is shown in Figure 3. As illustrated in the SDS‐PAGE image, a noticeable difference was observed in the electrophoretic patterns of the marinated beef samples and the nonmarinated group, as evidenced by the lower WBSF. Therefore, the myofibrillar protein fragments of all treated samples between 17 and 270 kDa partially or entirely faded during 24 hr of conditioning at cold storage, compared with the control, owing to the proteolytic activity of the exogenous/endogenous proteases. Thus, the intensity of the higher weighed proteins progressively diminished at different rates/amounts. Rawdkuen et al. (2013) pointed to the lower weighed protein bands produced at the bottom of the gel in the beef samples treated with the crude enzyme extract of *Calotropis procera* latex. Likewise, Maqsood et al. (2018) reported a 20‐kDa peptide band generated within the treatment of camel meat with bromelain, ficin, and papain. Similar reports were represented by Naveena et al. (2011) and Kim et al. (2013). The intensity and number of the protein bands higher than ~65 kDa showed a reduction, whereas elevation of the protein bands lower than ~25 kDa occurred. Among these enzymes, μ‐calpain, as an initiator of proteolysis, has a fundamental activity in postmortem proteolysis and meat tenderization. The main contractile proteins, namely, actin (45 kDa) and MHC (~220 kDa), underwent different degrees of degradation in marinated samples during aging. MHC is the main target tissue of broccoli/asparagus/ginger extracts and prolonged storage of the isolated myofibrillar proteins in the presence of these crude extracts could break down all the myofibrillar proteins (Ha et al., 2013). This function is apparently connected to the activity of the cysteine and aspartic proteases of crude extracts, calpain and cathepsin enzymes, as well as the synergistic effects of them during extended cold storage, which affects the protein structure to make the marinated beefsteaks softer and tender. Desmin (50 kDa) is the major intermediate filament in the skeletal muscle cells, which is vital in the maintenance of the cytoskeletal integrity; it was relatively hydrolyzed in marinated samples over a period of 3–24 hr compared to control (Tsai et al., 2012). The hydrolysis of these proteins has been revealed to decompose the muscle fiber structures with an associated decrease in shear resistance, texture cohesiveness, and an improvement in the meat tenderness during the postmortem storage (Kemp et al., 2010). Besides, the regulatory protein, that is, tropomyosin (33–35 kDa) band, became less intense in the marinated samples over 24 hr of storage while it was clearly identified in the unmarinated specimen. To conclude, the degradation of contractile, structural, and regulatory proteins in large amounts was more obvious in the marinated samples over the first 24 hr of storage than the control group.

### 3.3 Effect of marinating time on ultrastructural attributes of beef meat exposed to the marinade mixture

**FIGURE 4:**
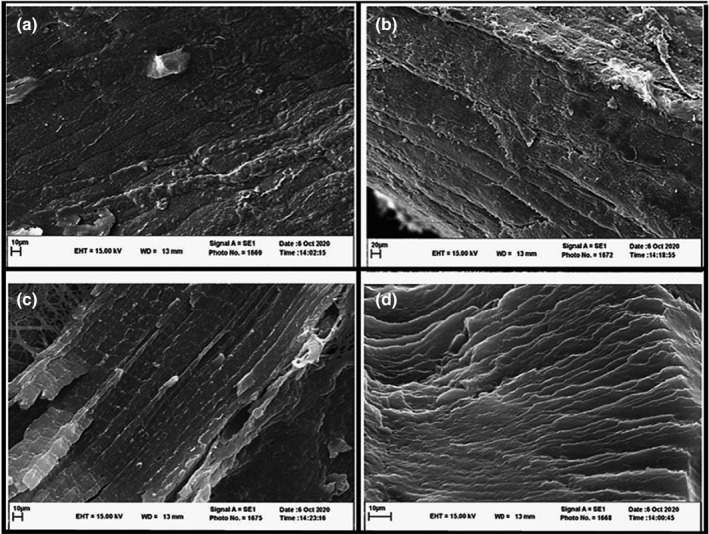
Scanning electron microscopy images of the longitudinal section of control and marinated raw beef meat cuts within 24 hr of marinating time (×1,000). Treatments: a: nonmarinade sample, b: marinated sample at 0 hr, c: marinated sample at 3 hr, d: marinated sample at 24 hr of storage at cold condition

Scanning electron microscopy images were employed to visualize the meat ultrastructure, particularly the arrangement of muscle fibers and their bundles, that the level of integrity and compactness determine meat tenderness. The effect of marinating time on ultrastructural attributes of beef meat is displayed in Figure 4. As shown in Figure 4, filaments of the nonmarinated sample were pressed firmly together, which seemed to be the lack of rupture results in toughness in the control group, while the SEM images of the marinated samples revealed significant spaces between muscle fibers and looser structure implying meat tenderness due to degradation and decomposition caused by marinade compared with control. These spaces between muscle filaments may control the moisture, including natural and/or absorbed water being immobilized by the muscles (Yogesh et al., 2012). Thereby, WHC enhancement in the marinated treatments could be due to the disintegration of muscle ultrastructure and solubilization of connective tissues in low pH conditions, as revealed by SEM images in this study. Hence, the existence of a direct correlation between WHC and soluble collagen as well as an indirect correlation between moisture content and WBSF value are properly confirmed by SEM images. Meanwhile, these changes may result in higher WHC, moisture content, soluble collagen, and lower WBSF values, suggesting the increased tenderness. The loss of connective tissues can be associated with the higher connective tissues, that is, perimysium, endomysium, and collagen protein dissolving due to enzymatic hydrolysis of meat proteins by natural and added proteases of broccoli/asparagus/ginger extracts, as well as the existence of acidic conditions served by soy sauce and vinegar. Similar observations have been documented in previous studies (Kim et al., 2013; Mazaheri Kalahrodi et al., 2020; Naveena et al., 2011).

## 4 CONCLUSION

Increased marinating times of 24 hr were found to produce more tender beef meat with increased WHC, soluble collagen, moisture contents, and decreased required force for shearing marinated beef meat cuts. These results have highlighted the efficiency of a short marinating time of 24 hr to accomplish the reduced level of required shear force and high level of moisture maintenance, which resulted in high tenderness and customer acceptance. To conclude, a strong positive correlation with the drip loss (*p* < .01) and a significant negative correlation with the moisture content (*p* < .01) were affected the tenderness of marinated beef meat. Furthermore, the electrophoresis patterns depicted a significant breakdown of MHC, desmin, actin, and tropomyosin during the first day of conditioning. Noticeable ultrastructural destruction and connective tissue proteolysis were recognized by microscopy images. These findings were a practical tenderness predictor to be employed for retailers and industrial producers.

## CONFLICT OF INTEREST

The authors declare no conflict of interest.

## ETHICAL APPROVAL

This study does not involve any human or animal testing.

## Data Availability

The data that support the findings of this study are available from the corresponding author upon reasonable request.
